# A Recent Insight Regarding the Phytochemistry and Bioactivity of *Origanum vulgare* L. Essential Oil

**DOI:** 10.3390/ijms21249653

**Published:** 2020-12-17

**Authors:** Adelina Lombrea, Diana Antal, Florina Ardelean, Stefana Avram, Ioana Zinuca Pavel, Lavinia Vlaia, Ana-Maria Mut, Zorita Diaconeasa, Cristina Adriana Dehelean, Codruta Soica, Corina Danciu

**Affiliations:** 1 Department of Pharmacognosy, “Victor Babes” University of Medicine and Pharmacy, Eftimie Murgu Square, No.2, 300041 Timisoara, Romania; lombrea.adelina@yahoo.com (A.L.); ioanaz.pavel@umft.ro (I.Z.P.); corina.danciu@umft.ro (C.D.); 2 Department of Pharmaceutical Botany, “Victor Babes” University of Medicine and Pharmacy, Eftimie Murgu Square, No.2, 300041 Timisoara, Romania; diana.antal@umft.ro (D.A.); ardelean.florina@umft.ro (F.A.); 3 Research Centre for Pharmaco-Toxicological Evaluation, “Victor Babes” University of Medicine and Pharmacy, Eftimie Murgu Square, No.2, 300041 Timisoara, Romania; cadehelean@umft.ro (C.A.D.); codrutasoica@umft.ro (C.S.); 4 Department of Pharmaceutical Technology, “Victor Babes” University of Medicine and Pharmacy, Eftimie Murgu Square, No.2, 300041 Timisoara, Romania; vlaia.lavinia@umft.ro (L.V.); mut.anamaria@umft.ro (A.-M.M.); 5 Research Center for Formulation and Technology of Drugs, “Victor Babes” University of Medicine and Pharmacy, Eftimie Murgu Square, No.2, 300041 Timisoara, Romania; 6 Department of Food Science and Technology, Faculty of Food Science and Technology, University of Agricultural Science and Veterinary Medicine, Calea Manastur, 3-5, 400372 Cluj-Napoca, Romania; zorita.diaconeasa@gmail.com; 7 Department of Toxicology, “Victor Babes” University of Medicine and Pharmacy, Eftimie Murgu Square, No.2, 300041 Timisoara, Romania; 8 Department of Pharmaceutical Chemistry, “Victor Babes” University of Medicine and Pharmacy, Eftimie Murgu Square, No.2, 300041 Timisoara, Romania

**Keywords:** *Origanum vulgare* L., essential oil, phytochemistry, bioactivity, pharmaceutical formulation

## Abstract

*Origanum vulgare* L. is a widely used aromatic plant, especially due to its content in essential oil, mainly rich in carvacrol and thymol. The ethnopharmacological uses of *Origanum vulgare* L. essential oil (OEO) comprise digestive, respiratory, or dermatological disorders. The review focuses on the increasing number of recent studies investigating several biological activities of OEO. The bioactivities are in tight relation to the phytochemical profile of the essential oil, and also depend on taxonomic, climatic, and geographical characteristics of the plant material. The antibacterial, antifungal, antiparasitic, antioxidant, anti-inflammatory, antitumor, skin disorders beneficial effects, next to antihyperglycemic and anti-Alzheimer activities were reported and confirmed in multiple studies. Moreover, recent studies indicate a positive impact on skin disorders of OEO formulated as nanocarrier systems in order to improve its bioavailability and, thus, enhancing its therapeutic benefits. The review brings an up to date regarding the phytochemistry and bioactivity of *Origanum vulgare* L. essential oil, underlining also the most successful pharmaceutical formulation used for skin disorders.

## 1. Introduction

*Origanum vulgare* L., commonly oregano, is one of the most renowned aromatic species, with a strong traditional background as a spice and medicinal plant, but also as a well-established source of valuable plant-based drugs in modern phytotherapy. This *Lamiaceae* plant is native to Europe, the North of the African continent, and most of temperate Asia, but the hotspot of its diversity is situated in the Mediterranean region and predominantly in Turkey [1]. It has also been introduced in North America, where it grows in several states along the East and West coast [2]. Commercial *O. vulgare* is either wild-collected or cultivated, with Turkey being the most important supplier of Mediterranean oregano. However, the common name of “oregano” is employed for several other species of the genus (*Origanum onites* or Turkish oregano, *O. majorana* or sweet marjoram, *O. minutiflorum* or Spartan oregano, *O. syriacum* var. *bevanii* or Israeli oregano) or even belonging to other genus (*Thymus, Satureja, Lippia, Coridothymus*) [3].

Botanically, *O. vulgare* is a perennial plant with a woody base and herbaceous stems, growing to a height of 20–80 cm. Its leaves are opposite, egg-shaped, 1–4 cm long, and 0.5–2.5 cm wide; the leaf margin is smooth and the tip varies from rounded to pointed. The flowers are relatively small, grouped in terminal and lateral paniculate inflorescences. Their corolla consists of 5 united petals, 0.4–0.8 cm long, having a white to purple color. The sepals are as well reunited; the inner part of the flower contains fours stamens and the pistil is constituted by two fused carpels. The fruits are small nutlets [4]. *Origanum vulgare* is a highly variable species, encompassing several subspecies, varieties, chemotypes, and hybrids. Six subspecies are recognized: *Origanum vulgare* subsp. *glandulosum* (Desf.) Ietsw., subsp. *gracile* (K.Koch) Ietsw., subsp. *hirtum* (Link) A.Terracc., subsp. *virens* (Hoffmanns. & Link) Ietsw., subsp. *viridulum* (Martrin-Donos) Nyman, and subsp. *vulgare* [2,5]. The first three subspecies are typical for the southern range of the species and considered to be of high quality and rich in volatile oils, as opposed to the last three subspecies vegetating in more Northern regions, and poorer in essential oils [6].

**Figure 1:**
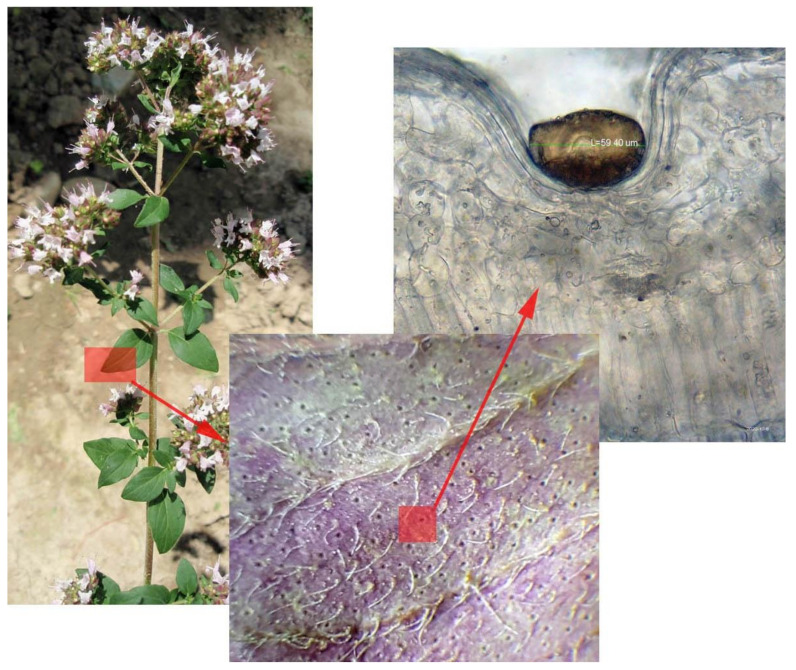
*Origanum vulgare*. Left image: flowering plant. The highlighted portion of the leaf surface is detailed in the middle image (short red arrow). Middle: Tectory and glandular trichomes on the lower leaf surface. The highlighted portion represents a secretory trichome, enlarged in the right image (long red arrow). Right: Peltate secretory trichome containing essential oil.

The essential oils, the most relevant constituents for the medicinal value of *O. vulgare*, are synthesized in peltate glandular trichomes, which occur on the surface of stems, leaves, and flowers (sepals, petals). These trichomes have an enlarged secretory head, made up of 12–16 glandulous cells covered by a common cuticle (Figure 1); volatile oils are released upon rupture of the cuticle. Except for these peltate hairs, much smaller capitate hairs also occur on both sides of the leaves and the epidermis of the kalyx. They have a 1–2 celled head and seem to be involved in the synthesis of other more hydrophilic metabolites like phenolic compounds and polysaccharides [7]. The factors that influence the density and size of glandular trichomes, and, thus, directly impact the yield in essential oil, are defined both genetically and environmentally. Greek oregano displays an increased density and size of peltate trichomes, in contrast to common oregano [8]. With increasing altitude, the frequency of peltate glands decreases, leading to the decline of the essential oil quantity. Interestingly, the chemical profile of the essential oil also varies with the altitude: in the Venetian region, lowland plants were shown to synthesize monoterpene and sesquiterpene hydrocarbon-rich essential oils, while high-altitude plants contained high amounts of aromatic and hydrocarbon monoterpenes and low amounts of sesquiterpenes [7]. Reduced water and nitrogen supply augment the density of peltate trichomes [9], while plant density does not affect the number of glandular hairs per leaf surface unit [10].

## 2. Traditional Uses

Uses of oregano in ethnomedicine have been linked to stomachic, carminative, expectorant, and emmenagogue properties [11]. It has been indicated as tinctures or teas in respiratory and digestive disorders, but also as ointments to treat wounds [12]. Its traditional uses include indigestions, diarrhea, cough, and bronchitis [13]. It has also been used as a remedy against pruritus, headaches, and depression [14]. Particular uses have been mentioned for different parts of the plant: while the aerial parts have been used in pain, cough, or sexual dysfunction, the seeds were used in urinary tract infections or menstrual disorders and the flowering branches were used externally by rubbing in place of fractures or to treat toothaches [15]. An ethnobotanical study showed that tea made from *Origanum vulgare* herb was traditionally used in Transylvania to treat sore throat [16]. In Turkey, the flowering branches and the leaves were prepared as infusions and used for cold, flu, headache, or toothache [17]. Arial parts of oregano represent a habitual spice, especial in Mediterranean countries [18]. In addition to culinary and medicinal uses, oregano was also used in perfumery or as flavoring for alcoholic beverages [19].

## 3. Chemical Composition

The aerial parts of *O. vulgare* contain essential oils with variable composition, as well as a diversity of flavonoids, tannins, phenolic glycosides, and terpenoids [20]. Luteolin-*O*-glucuronide and luteolin-7-*O*-glucoside are the main flavonoid derivatives found in the hydroalcoholic extracts, decoctions, and infusions of *O. vulgare*¸ while rosmarinic acid is the main phenolic acid [21]. The presence of caffeic acid, protocatechuic acid, vanillic acid, and *o*-coumaric acid has also been reported [22].

**Figure 2:**
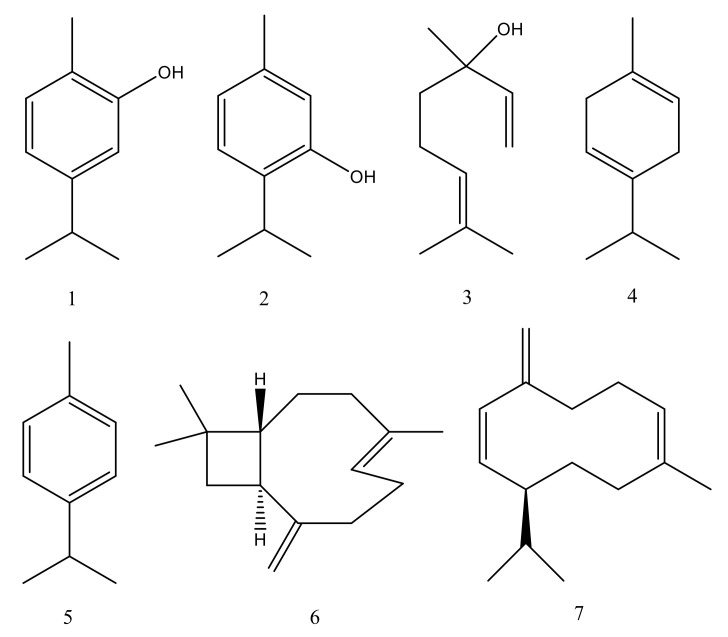
Chemical structures of main constituents of *Origanum vulgare* essential oil: (1) Carvacrol, (2) Thymol, (3) Linalool, (4) *γ*-Terpinene, (5) *p*-Cymene, (6) *β*-Caryophyllene, (7) Germacrene D.

The essential oil is the central type of oregano’s chemical constituents and has extensively been studied. As *O. vulgare* is a variable species, there are also variations in the chemical composition [23]. The volatile oil contains monoterpenes and sesquiterpene hydrocarbons, as well as phenolic compounds [14]. Terpenes such as thymol, carvacrol, *p*-cymene, *γ*-terpinene, and linalool are the main constituents (Figure 2). Depending on the major compounds, several chemotypes have been defined [24]. Investigation of 502 plants from 17 European countries led to the identification of three main *O. vulgare* chemotypes, depending on the proportions of acyclic linalool/linalyl acetate, cymyl and sabinyl compounds [23].

The essential oil composition varies according to environmental conditions, geographical area, harvesting time, and stage of plant maturity. The essential oil from plants in different countries has been evaluated in terms of chemical composition and major components have been identified depending on the area of origin [14]. Essential oils obtained from plants collected in Italy allowed the detection of 37 compounds, among which in greater quantities were thymol, carvacrol, linalyl acetate, and *γ*-terpinene [25]. Plants from Portugal revealed the presence of carvacrol, thymol, *γ*-terpinene, and *β*-fenchyl alcohol as main compounds in the essential oil [26], while the one from Montenegro had germacrene D, *β*-caryophyllene, linalyl acetate, and *α*-terpineol as major constituents [27]. The differences in the composition of essential oil in different organs of the plant have also been investigated, revealing variations in the content of certain compounds. For example, the stem oil was poorer in monoterpene hydrocarbons compared to leaf oil [28].

Plants with a higher essential oil content (*Origanum vulgare* subsp. *hirtum, Origanum vulgare* subsp. *glandulosum* and *Origanum vulgare* subsp. *gracile*) are characterized by the presence of carvacrol, thymol, *p*-cymene, and *γ*-terpinene, while the ones with lower content by the presence of sabinene, linalool, borneol, and sesquiterpenes [22]. The extensive investigation of *O. vulgare* essential oil led to the conclusion that the remarkable chemical variability of oregano can be explained by the up-/down regulation of the metabolic biosynthetic pathways. This research substantiated the fact that chemotype composition is in direct correlation with the climatic factors, with plants from the Mediterranean climate presenting an active cymyl- and/or linalool pathway, while plants growing in areas characterized by Continental climate are poorer in monoterpenes and display a more active sabinyl-pathway [23].

## 4. Pharmacological Activities

### 4.1. Antimicrobial Activity

The effectiveness of *Origanum vulgare* L. essential oil (OEO) against a wide range of pathogenic bacteria has been extensively studied. As it has been shown by an increased number of studies in the field, OEO represents an efficient alternative as an antimicrobial agent against both Gram-positive and Gram-negative bacterial strain [28,29,30]. Because essential oils are hydrophobic molecules, they have a greater permeability through the cell membrane and cause expansion of the cellular content. The death of the bacteria occurs by the drainage of crucial molecules and ions from the bacterial cell [29]. The disinfectant and antibacterial properties of the OEO were attested for the first time in ancient Greece, where it was frequently utilized for treating bacterial skin and wound contaminations. It was also used as a food preservative [30]. Carvacrol and thymol represent the two primary phenols, constituting almost 78–85% of the OEO and they are responsible for the plant’s antimicrobial properties [31]. The antibacterial activity of the OEO results from the high level of thymol which, according to Lambert et al. ties to membrane proteins and increases the permeability of the bacterial cell membrane [32]. In the same way, carvacrol acts on bacterial cells causing structural and functional damage, which increases bacterial cell membrane permeability [33]. The antibacterial activities explored for *Origanum vulgare* L. essential oil are represented in Table 1 for Gram-positive and Table 2 for Gram-negative bacteria.

Lu M. et al. investigated the efficacy of a commercial *Origanum vulgare* L. essential oil against multidrug-resistant (MDR) microbes such as methicillin-resistant *Staphylococcus aureus (MRSA)* and *Pseudomonas aeruginosa in vitro* and *in vivo*, in a mouse model of burn injury. OEO also exhibited comparable antibacterial activities against built-up biofilms formed by the 13 bacterial strains, with total inactivation of the biofilms of *Pseudomonas aeruginosa*, and *MRSA* at the concentrations of 1.0, and 0.4 mg/mL, respectively. Moreover, transmission electron microscopy (TEM) showed ultrastructural damages in *Pseudomonas aeruginosa* cells. They displayed a severe spillage of intracellular substances resulted from the contraction of the cell membrane after exposure to OEO for 1 h at 0.56 mg/mL. Noteworthy, the treatment of burn infection in BALB/c mice with OEO using a dose of 5 or 10 mg/mL, was a success. [37].

In a comprehensive study, Scandorieiro et al. described the synergistic and additive interactions of a two-drug combination of *Origanum vulgare* EO and green silver nanoparticles (bio-AgNP) produced by *Fusarium oxysporum* against multidrug-resistant bacteria such as methicillin-resistant Staphylococcus *aureus*, b-lactamase- and carbapenemase-producing, Escherichia *coli*, Escherichia *coli ESBL* and Escherichia *coli KPC.* In combination, OEO and bio-AgNP showed significantly lower minimum inhibitory concentration (MIC) values when compared with individual treatment (*p* < 0.05), where the two compounds together resulted in synergistic or additive antibacterial potential. Besides, the combination of OEO and bio-AgNP led to a faster reduction of CFU/mL than in individual treatment with bio-AgNP. Moreover, scanning electron microscope (SEM) revealed similar morphological alterations in non-methicillin-resistant Staphylococcus *aureus* cells exposed to three different treatments (OEO, bio-AgNP, and combination of the two), which appeared on cell surface blebbing after 6 h of treatment. Individual and combined treatments showed a reduction in cell density and a decrease in the exopolysaccharide matrix compared to untreated bacterial cells [35].

OEO with higher content in carvacrol and thymol, alone or in combination with silver nanoparticles, acts efficiently against Gram-positive and Gram-negative bacterial strains including multiresistant gram-positive and gram-negative bacteria *S. aureus*, *E. coli*, *P. aeruginosa, K. pneumoniae*.

### 4.2. Antifungal Activity

The antifungal activity of OEO is based on the presence of a high amount of thymol and carvacrol. Their antifungal action is related to the disturbance of the fungal cell wall integrity and with the interference of ergosterol synthesis [48]. Studies about the antifungal properties of Origanum vulgare L. essential oil are shown in Table 3.

### 4.3. Antiparasitic Activity

Relevant studies on the antiparasitic activity of OEO are shown in Table 4. This effect of OEO may be due to the presence of phenolic compounds (thymol and carvacrol) that interact with the permeability of the cytoplasmic cell membrane [53].

*Cryptosporidium parvum* is the second leading cause of persistent diarrhea among children living in poor households. Gaur et al. showed the potential anti-cryptosporidial effect of *Origanum vulgare* L. essential oil (origin: Turkey, steam extracted) in HCT-8 cells (Human colon adenocarcinoma). The immunodetection of infectivity using phase-contrast/fluorescent microscopy showed that OEO inhibited (*p* < 0.05) *Cryptosporidium parvum* growth in a dose-dependent manner: 55.6 ± 10.4% at 60 μg/mL OEO after 24 h incubation, without any apparent toxicity to the HCT-8 cells [54].

Santoro et al. explored the impact of the essential oil obtained from *Origanum vulgare* L. (harvested from Lavras, Brazil) on development and ultrastructure of *Trypanosoma cruzi*. Within 24 h incubation with OEO (from 25 to 250 μg/mL), the epimastigotes and trypomastigotes presented lysis of the cells and the IC_50_ values were 115μg/mL for trypomastigotes and 175 μg/mL for epimastigotes. Moreover, the examination of oregano-treated parasites (115 μg/mL) assessed by TEM showed slight morphological alterations in the plasma and flagellar membrane [55].

Pensel et al. demonstrated in vitro the effect of OEO (Buenos Aires Province, Argentina) against *Echinococcus granulosus* protoscoleces and cysts. The essential oil was added to the medium contained 10 μg/mL thymol. OEO diminished (*p* < 0.01) the viability of protoscoleces to 22.3 ± 1.2% after 60 days of incubation. TUNEL assay noticed DNA fragmentation and apoptosis of parasites treated with OEO for 16 h [56].

### 4.4. Antioxidant Activity

The antioxidant activity of *Origanum vulgare* L. essential oil is attributed due to the presence of carvacrol, thymol, and *p*-cymene, each one having the property to form chemical complexes with metal ions and free radicals (Table 5) [57]. The application of this bioactivity can be valued in food and pharmaceutical industries, as a safer alternative to the synthetic antioxidants [58].

### 4.5. Anti-Inflammatory Activity

OEO can inhibit the secretion of pro-inflammatory cytokines and also, down-regulates the expression of inflammatory genes due to the presence of carvacrol [65]. The anti-inflammatory activities studied for Origanum vulgare L. essential oil are shown in Table 6.

Cheng et al. determined the anti-inflammatory effect on lipopolysaccharide (LPS) murine macrophage cells (RAW264.7) after treatment with OEO (acquired from Meritech Bioengineering Co. Ltd. Guangzhou, China). ELISA commercial kits revealed the decreased levels of IL-1β, IL-6, and TNF-α after 12 h treatment with OEO (2.5, 5, 10 μg/mL). Chemiluminescence assay measured the ROS (reactive oxygen species) level using luminol. Moreover, pretreatment with OEO (2.5, 5, 10 μg/mL) restrained the ROS generation within the RAW264.7 cells in the presence of lipopolysaccharide. Noteworthy, LPS may initiate an inflammatory response through nicotinamide adenine dinucleotide phosphate (NADPH) oxidase activation. Furthermore, ROS production can be counteracted by inhibitors against NADPH oxidase. Therefore, the research team evaluated the NADPH oxidase activity of RAW264.7 cells treated with OEO, using ELISA kits. The results have shown that the OEO inhibited the lipopolysaccharide inflammatory response driven by NADPH oxidase and oxidative stress [67].

Laothaweerungsawat et al. tested a microemulsion of *O. Vulgare* essential oil (ME, 5% *w/w* OEO) on Raw 264.7 cells (murine macrophage cells). *Origanum vulgare* L. was harvested from Chaing Mai, Thailand and HPLC assay attested carvacrol as a major component of OEO. The microemulsion was investigated for the irritation properties in comparison with nonencapsulated OEO using HET-CAM (hen egg test-chorioallantoic membrane) assay. ME initiated a lower irritation score (IS = 3.1 ± 0.10) than nonemulsified OEO (IS = 4.8 ± 0.02) (*p* < 0.01). Furthermore, the ME showed higher inhibitory potential against IL-6 than the OEO (IC_50_ = 6.8 ± 2.0 and 15.5 ± 3.3 μg/mL, respectively (*p* < 0.05). Regarding the inhibition of TNF-α, ME had comparable inhibition to dexamethasone with the IC_50_ values of 5.4 ± 2.3 and 1.1 ± 0.9 μg/mL, respectively (*p* > 0.05) [68]. 

Carrasco et al. studied the anti-inflammatory activity of essential oils from 3 types of oregano harvested from Supra-Mediterranean and upper Meso-Mediterranean bioclimatic areas (Murcia, Spain). OEO samples were analyzed by GC-MS to determine their composition. (E)-*β*-Caryophyllene (0.5–4.9%), thymol (0.2–5.8%), *p*-cymene (3.8–8.2%), *γ*-terpinene (2.1–10.7%), and carvacrol (58.7–77.4%) were determined as main molecules. Sample from the Upper Meso-Mediterranean bioclimatic zone had the greatest inhibitory activity against lipoxygenase (LOX) (IC_50_ = 251.5 ± 1.4 µL EO/L) due to the presence of carvacrol and *γ*-terpinene as major compounds [69].

### 4.6. Antitumoral Activity

The potential therapeutic effects of plant essential oils in anticancer treatment has been inquired by many researchers in the field, due to the increased multidrug resistance and also the negative side effects of traditional chemotherapeutic agents [70]. The antiproliferative and cytotoxic effects of OEO have been demonstrated in the studies presented in Table 7.

Bioactive principles of OEO (purchased from Berjé USA) were evaluated for their apoptotic effects against human stomach cancer cell lines (AGS) by Balusamy et al. Antiproliferative property of the OEO in adenocarcinoma gastric cell line (AGS) was determined by MTT assay. After 48 h of incubation with OEO (5, 10, 25, 50 and 100 μg/mL), IC_50_ was 13.4 μg/mL. The best antiproliferative activity of OEO was found to be 100 μg/mL, where almost 100% of cells were induced by cell damage by OEO treatment. Apoptosis detection of stomach cancer cell lines treated with OEO was performed by Hoechst and PI staining. Hoechst staining showed typical apoptosis characteristic of cancer cells treated with 10, 25, 50, and 100 μg/mL, including darkly stained nuclei, nuclei shrinkage, and DNA fragmentation, segregated bodies, the formation of apoptotic bodies in the inner surface of the nuclei. Moreover, detection of OEO induced apoptosis using propidium iodide staining indicated that cells treated with 25 μg/mL and 50 μg/mL of OEO reduced cell viability and cell. Moreover, they studied the molecular mechanism associated with the mitochondrial-mediated apoptosis controlled by the B-cell lymphoma protein-2 (BCL-2) family members that stimulate pro-apoptotic proteins including Bcl-2-associated X protein (BAX). BAX expression gradually increased at the concentration of 10 μg/mL OEO (2.37-fold) and reached its maximum transcript accumulation at 50 μg/mL of 4.32-fold, respectively [72].

Elansary et al. published data regarding the anticancer activity of *Origanum vulgare* L. (northern Egypt) essential oil (50, 100, 200, 300, and 400 μg/mL) against several cancer cell lines using MTT assay: breast adenocarcinoma (MCF-7), cervical adenocarcinoma (HeLa), T-cell lymphoblast (Jurkat), colon adenocarcinoma (HT-29), and urinary bladder carcinoma (T24) and human cell line (HEK-293). Compared to positive controls (vinblastine sulfate and taxol) OEO presented significant antitumoral activities against MCF-7 (IC_50_ = 8.11 µg/mL, taxol-IC_50_ = 0.08 µg/mL), HeLa(IC_50_ = 13.41 µg/mL, vinblastine-IC_50_ = 2.5 µg/mL), Jurkat(IC_50_ = 27,05 µg/mL, vinblastine-IC_50_ = 0.1 µg/mL), HT-29(IC_50_ = 12.18 µg/mL, vinblastine-IC_50_ = 12.18 µg/mL), T24(IC_50_ = 105.5 µg/mL, vinblastine-IC_50_ = 63.31 µg/mL) cancer cell lines [38].

Carvacrol as a major phytocompound in OEO may be responsible, in part, for the antiproliferative potential of OEO, as indicated by others, acting as suppressor of kinase ERK1/2 (extracellular signal-regulated protein kinase1/2) and AKT (Protein kinase B) proteins while upregulating BAX and phosphor c-Jun N-terminal kinase (*p*-JNK) protein expression [18].

### 4.7. Beneficial Activity on Skin Disorders

The therapeutical proprieties of *Origanum vulgare* L. essential oil gained attention for skincare items and scientific research regarding its impacts on human skin cells. Recent approaches in this sense are presented in Table 8.

In a comprehensive study, Avola et al. investigated the biological effects of OEO (provided by Esperis S.p.A., Milan, Italy) in the restoring of the physiological cell homeostasis during wound and inflammation phenomena. To accomplish this, human keratinocyte cell line NCTC 2544 was used as an inflammatory in vitro model obtained by interferon-gamma (IFN-γ) and histamine stimulation or as a wound model attained by scratching the confluent monolayer of keratinocytes. The cultures treated with OEO showed a significantly lower level of ROS compared to the control. Furthermore, analysis regarding the amount of mRNA and proteins of ICAM-1, iNOS, and COX-2 were assessed by RT-PCR and Western blot. The results marked that the addition of OEO (25 µg/mL) for 72 h significantly reduced mRNA of ICAM-1, iNOS, and COX-2, suggesting that OEO enables cell homeostasis restoration during inflammatory events. Besides, keratinocytes stimulated with IFN-γ and histamine showed high levels of MMP-12 (a skin elastase) involved in the metabolism of elastic fibers and associated with the decrease in skin elasticity and consequent formation of wrinkles in various types of tissues during acute or chronic inflammatory disease. Compared to indomethacin, OEO (25 μg/mL) inhibited the MMP-12 expression to a higher degree. Evaluation by immunoblotting, under inflammatory condition in human keratinocyte cell line, showed the increased expression levels of proliferating cell nuclear antigen (PCNA), a marker of proliferation, whereas the amount of PCNA signal was significantly decreased by treatment with OEO (25 μg/mL). These results indicated that OEO acts as both an inducer of cell proliferation and a supporter of wound healing by PCNA modulation [73].

Laothaweerungsawat et al. revealed the cosmeceutical potential of two samples of *Origanum vulgare* L. essential oil as a skin-aging retarding agent. They compared OEO from Chiang Mai, Thailand obtained by hydrodistillation and a commercial oil purchased from Botanic essence (Product of Spain). Using ascorbic acid as positive control in anti-collagenase and anti-elastase activity determination and oleanolic acid as positive control in the determination of anti-hyaluronidase, the research group showed that OEO from Chiang Mai, possessed a significant higher anti-skin-aging activity compared to ascorbic acid (*p* < 0.01), with inhibition against collagenase, elastase, and hyaluronidase of 92.0 ± 9.7%, 53.1 ± 13.3%, and 16.7 ± 0.3%, at the concentration of 67, 25, and 4 µg/mL, respectively. However, the anti-hyaluronidase activity of both essential oils was mitigated when compared to oleanolic acid. The retardation of collagen and elastin loss may occur due to the phytocompound carvacrol identified in the highest amount in both essential oils OEO from a highland area of a tropical country (79.5%) and commercial OEO (64.6%) [63].

### 4.8. Effects on Melanin Production

Melanin biosynthesis is controlled by tyrosinase (polyphenol oxidase). An imbalance in the activity of this enzyme can lead to hyperpigmentation of the skin. Hydroxyl groups of phenolic phytocompounds in *Origanum vulgare* L. EO can bind the active site of the enzyme, which can inhibit enzymatic activity [75]. Moghrovyan et al. tested *Origanum vulgare* EO as a whitening agent for hyperpigmentation using colorimetric tyrosinase inhibition assay. The medicinal plant was collected from Gegharkunik province, Armenia. The values for tyrosinase inhibitory activity of *Origanum vulgare* EO and arbutin (positive control) were 26.5 ± 0.3% and 50.0 ± 0.1%, respectively [62].

Essential oils from *Origanum vulgare* subsp. *vulgare* (OVV) (Kesan-Edirne, Turkey) and *Origanum vulgare* subsp. * hirtum* (OVH) (Ermenek-Karaman, Turkey) were analysed by Sarikurkcu et al. for their inhibitory properties against the tyrosinase enzyme, which is responsible for melanin biosynthesis. The chemical composition of OVH was carried out by gas GC-FID and GC-MS techniques. The most abundant components were linalool (96.31%) and *β*-caryophyllene (1.27%). The anti-tyrosinase activity of the EOs was spectrophotometrically measured and the results were expressed as kojic acid equivalents (mgKAEs/g oil). *Origanum vulgare* subsp. *hirtum* was the most potent inhibitor with 45.60 mg KAEs/g oil when compared to OVV (8.30 mg KAEs/g oil). It was reported that linalool-rich essential oils could inhibit [75,76].

### 4.9. Hypoglycemic Activity

The inhibition of *α*-Amylase and *α*-glucosidase enzymes is an important strategy for maintaining blood glucose values. Essential oils extracted from 2 species of *Origanum vulgare* L.: *Origanum vulgare* subsp. *hirtum* from Ermenek-Karaman, Turkey, and *Origanum vulgare* subsp. *vulgare* from Kesan-Edirne, Turkey were investigated by Sarikuku et al. for their antidiabetic activity. The highest *α*-glucosidase inhibitory activity was recorded for *Origanum vulgare* subsp. *vulgare* with 6.04 mmol ACEs/g oil. As to *α*-amylase inhibitory activity, the EOs exhibited similar activity (0.14 for *Origanum vulgare* subsp. *hirtum* and 0.13 mmol ACEs/g oil for *Origanum vulgare* subsp. *vulgare*) [75].

### 4.10. Effects on Human Sperm Mobility

New data regarding the biological activities of *Origanum vulgare* L. essential oil was reported by Mbaye et al. The research team studied the impact of oregano (harvested from Fes, Marocco) essential oil on sperm’s motility and vitality. The mobility was assessed by a Computer Assisted Sperm Analysis and the evaluation of sperm vitality was performed by eosin 2% staining and analyzed via optical microscopy. Following 5 min of incubation OEO (range of concentrations: 10^−1^, 10^−2^, 10^−3^, and 10^−9^) gave promising values of mobility and vitality (73 ± 0.07% respectively 74 ± 0.07%) suggesting that OEO can be a therapeutic agent in qualitative abnormalities of human sperm [77].

Mbaye et al. continued to study *Origanum vulgare* L. essential oil (Fes, Marocco) on the advanced parameters of mobility and the integrity of the sperm DNA of 25 male infertile volunteers. The data obtained during experiments was the subject of a statistical study. The results of OEO (0.2% (*w*/*v*) sterile agar solution) supplementation on the characteristic parameters of mobility and nuclear quality were obtained by the Student *t*-test. The experiment has shown a significant stimulating effect on advanced mobility parameters: curvilinear velocity (*p* < 0.01), linear velocity (*p* < 0.01), mean path velocity (*p* < 0.01), and amplitude of displacement (*p* < 0.01) [78].

### 4.11. Anti-Alzheimer Activity

The ability of phytocompounds present in essential oils to inhibit acetylcholinesterase is interesting, as it can be used as possible treatment of some nervous illnesses such as Alzheimer’s disease [79]. Carrasco et al. tested *in vitro* cholinesterase inhibitory activity of essential oils obtained from 2 samples of *Origanum vulgare* L. grown in Supra Mediterranean and Upper Meso-Mediterranean bioclimatic regions in Murcia, Spain. Quantitative determination of the relative and chiral distribution of the main components of each EO was performed by fast gas chromatography/mass spectrometry (FGS/MS). Results showed that myrcene, *γ*-terpinene, and thymol were found in high proportion in OEO from Supra Mediterranean bioclimatic region, whereas high concentrations of 1,8-cineole, linalool, borneol, carvacrol, and *β*-bisabolene were found in OEO Meso-Mediterranean field. Concerning inhibitory the activity on acetylcholinesterase (AChE) *Origanum vulgare* L. grown in Supra Mediterranean region had the greatest inhibitory activity (IC_50_ = 73.7 ± 0.5 µL EO/L), followed by OEO from Meso-Mediterranean region (IC_50_ = 61.5 ± 0.5 µL EO/L) [69].

The acetylcholinesterase inhibitory effect of the EO obtained from 2 species of *Origanum vulgare* L.: *Origanum vulgare subsp. vulgare* (OVV) and *Origanum vulgare subsp. hirtum* (OVH) harvested from Turkey was reported by Sarikurcku et al. The EOs showed a similar action both on AChE (1.57 for OVH and 1.64 mg GALAEs/g oil for OVV) and BChE inhibitory activities (1.74 for OVH and 1.75 mg GALAEs/g oil for OVV). The research team analyzed the oils by GC-FID and gas chromatography/mass spectrophotometry GC-MS techniques. Thus, the inhibitory properties of OVV may be explained by the great amount of thymol and carvacrol, while the inhibitory activity of OVH on AChE and BChE may be due to the high amount of linalool [75].

## 5. Drug Delivery Systems for OEO Topical Application

**Figure 3:**
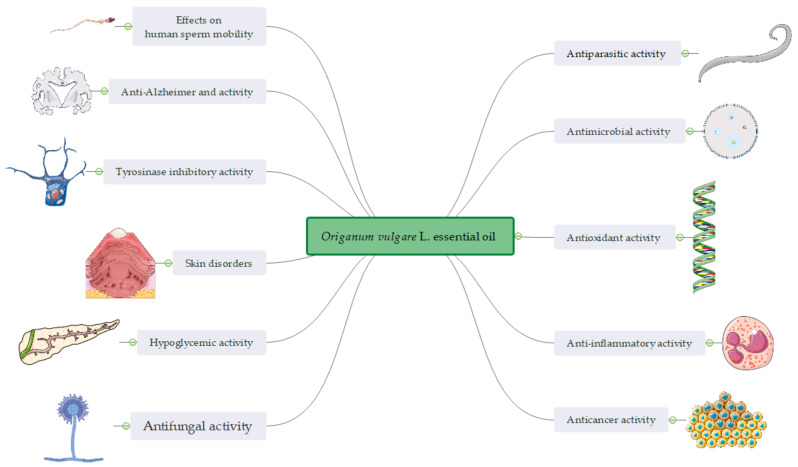
A snapshot of the bioactivity of *Origanum vulgare* L. essential oil.

Due to its multiple pharmacological activities, as shown in Figure 3, as reviewed above, but especially due to its antimicrobial, anti-inflammatory, and antitumor effects, oregano essential oil can be used as natural alternative to synthetic drugs, being a promising candidate for therapy of various conditions with microbial, inflammatory and tumor etiology [80]. Although, nowadays oregano essential oil is frequently used in traditional medicine and several “Do It Yourself” recipes are available, there is a lack of pharmaceutical preparations containing this essential oil mainly because, like other volatile oils, its incorporation into pharmaceutical topical dosage forms, especially hydrophilic ones, being confronted to technological limitations. These constraints are determined by the hydrophobic, volatile, and reactive nature of oregano essential oil bioactive components [81]. However, in the recent years some attempts were made to incorporate this volatile oil as active ingredient in different nanosystems (nanoparticles, nanoemulsions, and microemulsions) as colloidal drug carriers. 

OEO was encapsulated into chitosan-Tween 80 nanoparticles prepared via a two-step method, consisting in the formation of an oil-in-water emulsion, followed by ionic gelation. The OEO-loaded chitosan nanoparticles showed regular distribution, spherical shape, an average diameter ranging from 309.8 nm to 402.2 nm and the loading capacity and encapsulation efficiency in the range of 1.32–2.12% and 5.45–24.72%, respectively. By increasing the OEO content of chitosan-nanoparticles from 0.1 to 0.8 g/g chitosan, their loading capacity increased, whereas their encapsulation efficiency decreased. The in vitro release profile of OEO from chitosan-nanoparticles indicated an initial burst effect (at low OEO concentration) followed by a subsequent slower release (at higher OEO concentrations) and suggested their potential as controlled release nanosystems for OEO [82].

Taleb et al. reported the development of a pharmaceutical oil-in water (O/W) nanoemulsion for dermal delivery of OEO to improve its poor water solubility and, thus, extending the application of OEO in aqueous formulations with antimicrobial activity useful in acne treatment. In this study, OEO was selected as active ingredient of the nanoemulsion, because among all tested EO, it exhibited the highest antimicrobial effect against acne-causing bacteria in vitro [39]. The topical O/W nanoemulsion consisting of 95% (*w*/*w*) water and 5% (*w*/*w*) mixture of OEO and Pluronic F127 (0.5% essential oil and 4.5% of Pluronic F127) was prepared by a low energy method at room temperature. The obtained O/W nanoformulation presented a low size distribution indicated by a particle size of 39.54 nm and a polydispersity index of 0.285, and remained clear and stable for four weeks at ambient temperature, without any signs of cloudiness, creaming, or phase separation. Following in vivo epicutaneous application of the proposed OEO nanoemulsion on a standardized acne mouse model, both inflammation and bacterial load decreased significantly and the tissue healing was superior in comparison with the 2% erythromycin solution used as a positive control. Authors suggested that antibacterial effects of OEO, like those of other essential oils [83], were improved by its formulation as O/W nanoemulsion and proposed the developed OEO nanoemulsion as new natural and effective alternative anti-acne treatment to avoid the drawbacks associated with the often-prescribed antibiotics.

Laothaweerungsawat et al. investigated the effect of OEO encapsulation in microemulsion-based systems on in vitro transdermal delivery of carvacrol, the major component of this essential oil, possessing analgesic and anti-inflammatory activity [68]. Microemulsion systems containing OEO were successfully obtained using Tween 60 and butylene glycol as surfactant and co-surfactant, respectively. From the pseudoternary phase diagram constructed using water dilution method, three microemulsion formulations were selected and evaluated for different physicochemical properties, including appearance, particle size and size distribution, zeta potential, viscosity, pH, and stability. Furthermore, irritation potential of OEO microemulsions (expressed as Irritation Score) versus that of a 5% (*w*/*w*) OEO solution in butylene glycol were investigated using the hen’s egg test. Transdermal absorption and skin maintenance of carvacrol from the formulation was studied *in vitro*. Finally, the IL-6 and TNF-α secretion was determined to evaluate the anti-inflammatory activity of OEO containing microemulsions. According to the results of physicochemical characterization, the optimal formulation was considered the microemulsion containing 5% OEO as oil phase and active component, 25% Tween 60 as surfactant, 25% butylene glycol as cosurfactant, and 45% deionized water. With the narrowest polydispersity index (0.30 ± 0.07) and the lowest surfactant matter, this formulation of OEO displayed the smallest droplet size (179.5 ± 27.9 nm). The skin irritation effect of OEO was reduced to a large extent by this formulation (IS = 3.1 ± 0.10 versus IS = 4.8 ± 0.02 of blank formulation and IS = 5.0 ±0.01 of 5% OEO solution). Furthermore, this OEO microemulsion released carvacrol in a sustained manner, delivered 6.5 times more carvacrol through the skin than the OEO solution and produced the retention of a significant amount of carvacrol in the skin layer (2.6 ± 1.3%). The OEO anti-inflammatory activity was greatly improved by its incorporation in this microemulsion, which exhibited more potency in inhibiting IL-6 secretion and a comparable inhibitory effect on TNF-α secretion to that of dexamethasone [68].

## 6. Conclusions

*Oregano vulgare* L. is an important Mediterranean aromatic plant of great value in traditional phytotherapy, next to its widespread dietary uses. Ethnopharmacological recommendations associated to the use of oregano include digestive, respiratory, and dermatological complaints. Owing to the content of carvacrol and thymol rich essential oils, the major associated pharmacological effects are the antimicrobial properties. This review was tailored to cover important details regarding the source of *Origanum vulgare* L. plant material, focusing on the phytochemistry of the essential oil content, linking it to the lately investigated bioactivities, but also to current approaches in dermal drug carrier systems using OEO. 

*Origanum vulgare* is a highly variable species. The important variability of the essential oil composition depends on climate, environmental conditions, and geographical area. The phytochemical profile of OEO was extensively studied, and three main *O. vulgare* chemotypes were established, depending on the proportions of acyclic linalool/linalyl acetate, cymyl, and sabinyl compounds. The most investigated OEO are characterized by the presence of higher concentrations of carvacrol, thymol. Still, standardization of OEO according to main compounds is of great importance for relevant correlation with its biological activity. 

OEO particularly high in carvacrol and thymol represents an efficient alternative as antimicrobial agent against both Gram-positive and Gram-negative bacterial strains including multiresistant Gram-positive and Gram-negative bacteria *S. aureus*, *E. coli*, *P. aeruginosa*, *K. pneumoniae*. The antioxidant activity of OEO is attributed to the presence of the same compounds. OEO decreases the expression of pro-inflammatory cytokines and of inflammatory genes, due to the presence of carvacrol, emphasizing multiple mechanisms of action. The antitumor effects of OEO were reported for several types of cancer, including breast, colon, hepatic, or cervical cancer, with IC50 values ranging approximatively from 8 to 300 µg/mL. 

Furthermore, some of the common skin disorders such as acne, wound repair, or aging, were also shown to benefit from the antibacterial, anti-inflammatory, and antioxidative properties of OEO. The inhibition of hyaluronidase, collagenase, and elastase are correlated especially to the carvacrol content, while the antimelanin effect by tyrosinase inhibition was observed for linalool-rich essential oil type.

Only in recent years few promising approaches in the OEO use as pharmaceutical preparations have been reported. Mainly referring to dermal formulations, as topical application of OEO they were proven to be effective, generally safe and useful in various skin conditions. From our knowledge, nanocarriers of two categories, namely polymeric nanoparticles and lipid carriers including nano- and microemulsions, were proposed as drug delivery systems for OEO. OEO nanoencapsulation in such colloidal drug carriers is attractive because it allows to overcome the major disadvantage of OEO use, by decreasing its volatility, improving its stability and hydro solubility, and enhancing its bioavailability and therapeutic efficacy. 

OEO is a highly valuable source of active phytocompounds with important biological effects. Beside the well depicted biological effects, reviewing the literature leads to the conclusion that there is a need of future preclinical and clinical studies, oriented towards the development of topical OEO-loaded nano delivery systems with improved bioavailability and important antimicrobial, anti-inflammatory, antitumor, and wound-healing effects.

## Figures and Tables

**Table 1:** Antibacterial activity against Gram-positive bacteria.

Bacterial Strain	Dose	Details about the Source of the Oil and Tested Concentration	Reference
*Staphylococcus aureus*	MLC (minimal lethal concentration) = 320 μg/mL CZD (clear zone diameter) = 35 mm	Cold-pressed OEO was purchased from a local market in Mecca, Saudi Arabia (100 µL) Using TLC they have identified 91% lipids, followed by 0.7% glycolipids and 0.5% phospholipids. The research team also identified high amounts of linoleic and oleic acids. Both fatty acids accounted for 83% of the total fatty acid methyl esters. HPLC assay confirmed high levels of *α*-, *β*-, *γ*- and *δ*-tocopherols in OEO 180.4, 60.4, 650 and 117.6 mg/100 g oil, respectively. Besides, amounts of *α*-, *γ*- and *δ*-tocotrienols were 521, 58.9, and 430 mg/100 g oil, respectively. -MLC and CZD values were determined by agar well diffusion method Standards for comparison in antibacterial tests were Augmentin (30 µg), Chloramphenicol (30 µg), with CZD values 40 mm and 25 mm, respectively.	[34]
	MIC(minimum inhibitory concentration) = 0.596 mg/mL MBC (minimum bactericidal concentration) = 0.596 mg/mL MRSA MIC = 1.193 mg/mL MBC = 1.193 mg/mL	OEO was obtained from Ferquima Industry and Commerce of Essential Oils (São Paulo, Brazil) The main components consisted of: carvacrol (71%), thymol(3%), gamma-terpinene(4.5%), para-cymene(3.5%), beta-caryophyllene(4%) Tested concentrations of OEO ranged from 0.075 to 9.540 mg/mL MIC and MBC were determined by the broth dilution method	[35]
	CZD = 41.025 mm (ATCC 43300) CZD = 32.50 mm (ATCC 29213)	*Origanum vulgare ssp. hirtum* was collected from Kürtün, Turkey GC-MS/FID attested the presence of: carvacrol(65.080 ± 0.003%), thymol(10.490 ± 0.003%), *γ*-terpinene(7.340 ± 0.003%) Antibacterial activity of the essential oil was evaluated by disc diffusion method	[36]
	MIC = from 0.08 to 0.16 mg/mL A normal lessening in bacterial luminescence of 2.9 log10 was accomplished in 40 min at 5 mg/mL of OEO	OEO was acquired from Bulk Apothecary (Aurora, CO, USA) By the help of GS-MS analysis the main phytochemicals have been identified: carvacrol (72.25%) thymol (6.62%), *p*-cymene (5.21%), *γ*-terpinene (4.12%), *α*-pinene (1.21%) For determination of MIC, broth microdilution assay was performed. For the antibiofilm activity of the EO, Alamar Blue assay was employed Real-time monitoring of disease within the mouse burn wounds in vivo was performed through bioluminescence imaging.	[37]
	MIC = 0.28 mg/mL MBC = 0.67 mg/ml	*Origanum vulgare* L. was harvested from Alexandria, Behera and Matrouh in northern Egypt. GS-MS assay detected OEO’s main constitutes: pulegone 77.45%, menthone 4.86%, cis-isopulegone 2.22%, piperitenone 2.13%. The antibacterial activity of the essential oil was estimated using the microdilution method	[38]
*Staphylococcus epidermidis*	CZD = 52.00 mm	Source of the *Origanum vulgare* L. and chemical constituents were mentioned above in reference [35].	[36]
	MIC = 0.67 mg/mL MBC = 1.34 mg/mL	OEO was provided from the Department of Food Science and Technology, Nebraska University, Lincoln, NE, USA Chemical components of OEO were analyzed using GC-MS and showed thymol 99.44%, *p*-cymene, cineole and *γ*-terpinene as major constituents. OEO was screened for the antibacterial activities using broth microdilution method.	[39]
*Streptococcus pyogenes* Erythromycin-resistant Group A *Streptococcus pyogenes*	MIC = 256 to 512 μg/mL	OEO was purchased from Sigma–Aldrich (St. Louis, MO, USA) Phytochemical constituents are not mentioned MIC was determined by agar dilution and microdilution methods.	[40]
	MIC = 0.5 mg/mL MBC = 0.5 mg/mL MBEC(minimal biofilm eradication concentration) = 0.5 mg/mL MBIC(minimum biofilm inhibitory concentration) = 0.5 mg/mL OEO at the concentration of 0.5 mg/mL, caused 99.9% elimination of the initial bacterial inoculum after 0.08 h (5 min) of exposure	Oregano (*Origanum vulgare* L.) was collected from Truro, NS, Canada. According to the GC-FID(gas chromatography-flame ionization detector), carvacrol (91.6%) was the main phytoconstituent in OEO The effects of the OEO on the inhibition of bacterial growth was determined using a micro- broth dilution method. The bactericidal activity of the OEO was studied using modified time-to-kill assays.	[41]
*Streptococcus pneumoniae*	MIC = 2.5–10 μL/mL The treated biofilm with EO showed a significant reduction in the number of adherent bacteria and also the size of aggregates, which were reduced to small clusters or even single cells. OEO was able to significantly reduce biofilm formation as well as eradicate preestablished biofilms (*p* < 0.05).	*Origanum vulgare* L. was obtained from Tehran, Iran. GS-MS (gas chromatography-mass spectrometry) analysis showed for OEO, the main compounds were: pulegone (44.31%), 1,8-cineole (17.47%), borneol (6.20%). MIC was determined by broth micro- dilution method. The anti-biofilm activity of OEO (tested concentrations were MIC/2, MIC/4, and MIC/8) was determined by Microtiter-Plate Test and SEM (scanning electron microscope).	[42]
*Bacillus cereus*	MIC = 1.56 µL/mL MBC = 3.125 µL/mL	*Origanum vulgare* L. was collected from Latakia, Syria. The essential oil was analyzed by GC-MS and the major components were: terpinen-4-ol (24.90%), gamma-terpinene (10.57%), o-cymene (8.90%) Tested concentrations ranged from 200 to 0.0487 µL/mL	[43]
	MIC = 0.11 mg/mL MBC = 0.21 mg/mL	*Origanum vulgare* L. was harvested from Alexandria, Behera and Matrouh in northern Egypt. GS-MS assay detected OEO’s main constitutes: pulegone (77.45%), menthone (4.86%), cis-isopulegone (2.22%), piperitenone (2.13%) The antibacterial activity of the essential oil was estimated using the microdilution method.	[38]
*Enterococcus faecalis*	MIC = 8 mg/mL Inhibition zone = 13 ± 1 mm	OEO was obtained from the Magnolia Company (Tehran, Iran) The chemical composition of OEO was determined by GC–MS and the main constituents were: terpinene-4-ol (21.43%), g-Terpinene (12.32%), carvacrol (11.67%) thymol (9.45%) MIC value was determined by the broth microdilution method. Disk diffusion was employed for estimating the antibacterial activity of 20 µL of the Eos.	[44]
	CZD = 24.25 mm	Source of the *Origanum vulgare* L. and chemical constituents were mentioned above in reference [35].	[36]
*Listeria monocytogenes*	MIC = 0.6 µL/mL- L. monocytogenes ATCC 7644, LM17 MIC = 1.2 µL/mL- L. monocytogenes LM 4	The OEO used in this study was purchased from an Italian company: Zuccari SRL (Trento) GC-MS attested the presence of: carvacrol (68,1%), o-Cymene (5,9%), -thymol (3,7%) MIC values were obtained according to the microdilution method.	[45]
	MLC = 320 μg/mL CZD = 15 mm	Source of the *Origanum vulgare* L. and chemical constituents were mentioned above in reference [34].	[34]
	MIC = 0.40 mg/mL/ MBC = 0.83 mg/mL	Source of the *Origanum vulgare* L. and chemical constituents were mentioned above in reference [38].	[38]
*Propionibacterium acnes*	MIC = 0.34 mg/mL MBC = 0.67 mg/mL	Source of the *Origanum vulgare* L. and chemical constituents were mentioned above in reference [39].	[39]

**Table 2:** Antibacterial activity against Gram-negative bacteria.

Bacterial Strain	Dose	Details about the Source of the Oil and Tested Concentration	Reference
*Escherichia coli*	MLC = 160 μg/mL CZD = 33 mm	Source of the *Origanum vulgare* L. and chemical constituents were mentioned above in reference [34].	[34]
	MIC = 0.596 mg/mL MBC = 0.596 mg/mL	Source of the *Origanum vulgare* L. and chemical constituents were mentioned above in reference [35].	[35]
	CZD = 29 mm	Source of the *Origanum vulgare* L. and chemical constituents were mentioned above in reference [36].	[36]
	MIC = 0.25 mg/mL MBC = 0.58 mg/mL	Source of the *Origanum vulgare* L. and chemical constituents were mentioned above in reference [38].	[38]
*Pseudomonas aeruginosa*	MIC = from 0.32 to 0.64 mg/mL A normal lessening in bacterial luminescence of 2.9 log10 and 3.5 log10 were accomplished in 60 min at a concentration of 5 or 10 mg/mL of OEO. Furthermore, they didn’t take notes of any sign of reinfection within the following days in OEO-treated mice.	Source of the *Origanum vulgare* L. and chemical constituents were mentioned above in reference [37].	[37]
	MIC = 0.15 mg/mL MBC = 0.34 mg/mL	Source of the *Origanum vulgare* L. and chemical constituents were mentioned above in reference [38].	[38]
*Klebsiella pneumoniae* *Klebsiella oxytoca*	MIC = 73.5 mg/mL MIC = 0.9 mg/mL	*-Origanum vulgare* L. was harvested from Thessaloniki, Greece Phytochemical constituents were not mentioned MIC of the OEO was determined by broth microdilution method.	[30]
*Helicobacter pylori*	*Origanum vulgare subsp. vulgare* essential oil-MIC = 2 μL/mL *Origanum vulgare subsp. hirtum*-MIC = 2 μL/mL	*-Origanum vulgare subsp. vulgare and Origanum vulgare subsp. hirtum* were collected from Vojvodina, Serbia. GS-MS analysis attested the presence of monocyclic monoterpenes (89%), mostly phenolic carvacrol (71%) followed by *γ*-terpinene (8.36%) in *Origanum vulgare subsp. hirtum* essential oil and a high content of sesquiterpenes (58.0%), mostly caryophyllene oxide being the single most abundant component of *Origanum vulgare subsp.vulgare* essential oil.	[46]
*Salmonella enteritidis*	MSC after 18 h of incubation = 130 μg/mL	OEO was purchased from Ferquima-Industry and Trade Essential Oils Phytochemical constituents were not mentioned. Bacterial expressed proteins were assessed by 2D-SDS-PAGE. 2D-SDS-PAGE revealed a stress response with differential expressions of chaperones and cellular protein synthesis was intervened by the bacterial flagging system. Antibacterial inhibitory activity of the OEO was related to the presence of thymol and the research team observed an irregularity in DNA synthesis.	[47]
	MLC = 160 μg/mL CZD = 30 mm	Source of the *Origanum vulgare* L. and chemical constituents were mentioned above in reference [34].	[34]

**Table 3:** Antifungal activity of *Origanum vulgare* L. essential oil (OEO).

Fungal Strain	Dose	Details about the Source of the Oil and Tested Concentration	Reference
*Candida albicans*	MIC_50_ = 200 µg/mL MIC_90_ = 200 µg/mL MIC range = 150–250 µg/mL	*Origanum vulgare. ssp. vulgare* in the pre-flowering stage was collected in Piranshahr, Azarbaijan. Fungal strains were collected from mucosal layers of the oral cavity in HIV-positive patients. The in vitro antifungal effectiveness was performed using microdilution and disc diffusion methods. According to GC-FID and GC-MS analysis, the main components of OEO were: thymol (27.3%), *γ*-terpinene (20.7%), carvacrol (16.1% Fungal strains were collected from mucosal surfaces of the oral cavity of oropharyngeal candidiasis in HIV-positive patients.	[48]
	OEO 1%, after 48 h incubation, PZ(precipitation zone) = 0.90 OEO 5%, after 48 h incubation, PZ = 0.95 OEO 10%, after 48 h incubation, PZ = 0.97	*Origanum vulgare* L. was collected from Chile. GC-FID identified the major constituents of OEO: thymol (21.95%) carvacrol (4.71%), *p*-cymene (1.13%), *γ*-terpinene (2.43%) limonene (2.59%) The enzymatic assay tested phospholipase anti-enzymatic properties. The results were analyzed at 24, 48, 72, and 96 h by measuring the precipitation zone (PZ). The OEO at 1%, 5% and 10% presented significant reductions in the production of the phospholipase enzyme produced by *Candida albicans* strains.	[49]
	MIC = 0.26 mg/mL MFC(minimum fungicidal concentration) = 0.63 mg/mL	Source of the *Origanum vulgare* L. and chemical constituents were mentioned above in reference [38].	[38]
	MIC_50_ = 250 mg/L MIC_90_ = 500 mg/L MFC_50_ = 500 mg/L MFC_90_ = 500 mg/L	*Origanum vulgare* L. was collected from Lublin, Poland. According to GS-MS analysis, OEO contained carvacrol (57.3%), *γ*-terpinene (24.3%), *p*-cymene (12.5%), Antifungal activity against clinical isolates of oral *Candida albicans* was evaluated using the broth microdilution method	[50]
*Trichophyton rubrum* *Trichophyton* *mentagrophytes*	MLC = 40 μg/mL CZD = 38 mm MLC = 40 μg/mL CZD = 42 mm	Source of the *Origanum vulgare* L. and chemical constituents were mentioned above in [33]. Standards for comparison in antifungal tests were Nystatin BP, 100 μg/mL, with CZD = 38 mm and Fluconazole, 100 μg/mL with CZD = 34 mm (Trichophyton rubrum) Standards for comparison in antifungal tests were Nystatin BP, 100 μg/mL, with CZD = 40 mm and Fluconazole, 100 μg/mL with CZD = 35 mm (Trichophyton mentagrophytes).	[34]
*Aspergillus flavus*	MLC = 320 μg/mL CZD = 36 mm	Source of the *Origanum vulgare* L. and chemical constituents were mentioned above in reference [33]. Standards for comparison in antifungal tests were Nystatin BP, 100 μg/mL, with CZD = 40 mm and Fluconazole, 100 μg/mL with CZD = 38 mm.	[34]
	MIC = 100 µg/mL, MFC = 100 µg/ml	*Origanum vulgare ssp. gracile* was harvested from Iran. Phytochemical constituents were not mentioned. MIC and MFC values were obtained by agar disk diffusion and microwell dilution methods.	[51]
	MIC = 0.16 mg/mL, MFC = 0,35 mg/mL	Source of the *Origanum vulgare* L. and chemical constituents were mentioned above in reference [38].	[38]
*Malassezia furfur*	MIC = 780 µg/mL	Source of the plant was not mentioned OEO was characterized by GC and GC-MS and presented high contents of thymol (45.43%) and *ɣ*-Terpinene (23.69%). MIC against Malassezia furfur strains that had shown resistance to fluconazole was measured according to the broth microdilution protocols.	[52]
*Penicillium funiculosum* *Penicillium ochrochloron*	MIC = 0.25 mg/mL MFC = 0.61 mg/mL MIC = 0.33 mg/mL MFC = 0.71 mg/mL	Source of the *Origanum vulgare* L. and chemical constituents were mentioned above in reference [38].	[38]

**Table 4:** Antiparasitic activity of *Origanum vulgare* L. essential oil (OEO).

Parasite	Dose	Details about the Source of the Oil and Tested Concentration	Reference
*Cryptosporidium parvum*	At 60 μg/mL OEO reduced *Cryptosporidium parvum* infectivity to 55.6 ± 10.4%	OEO (origin: Turkey, steam extracted) was obtained from Oregano World, Hollywood. HPLC analysis of the OEO attested the presence of carvacrol (594.6 ± 10.0 μg/mL) Tested concentrations of OEO were: 0, 7, 15, 30, 60, 125, 250, 500 and 1000 μg/mL Infectivity was assessed via immunofluorescence detection using phase-contrast/fluorescent microscopy.	[54]
*Trypanosoma cruzi*	IC_50_ = 175 μg/mL—inhibited epimastigote growth IC_50_ = 115 μg/mL—induced trypomastigote lysis	*Origanum vulgare* L. was harvested from Lavras, Brazil. Qualitative and quantitative determination of the major phytocompounds were identified using GS-MS and GS-FID: 3-cyclohexen-1-ol (26.2%), *γ*-terpinene (16.0%), *α*-terpineol (12.3%) Concentrations of the OEO ranged from 25 to 250 μg/mL IC_50_ was determined after 24 h incubation by cell counting.	[55]
*Echinococcus granulosus*	OEO (10 μg/mL) diminished (*p* < 0.01) the viability of protoscoleces to 22.3 ± 1.2% after 60 days of incubation	*Origanum vulgare* L. was harvested from Buenos Aires, Argentina Qualitative and quantitative determination of the major phytocompounds were identified using GS-MS and GS-FID and confirmed the presence of: carvacrol (20.14%), thymol (19.71%), *γ*-terpinene (12.77%) *In vitro* viability was assessed by the methylene blue exclusion test.	[56]

**Table 5:** Antioxidant activity of *Origanum vulgare* L. essential oil (OEO).

Method of Study	IC_50/_Antioxidant Activity (%)	Details: Source, Phytochemical Composition; Formulation	Reference
2,2′-Diphenyl-1-Picrylhydrazyl-(DPPH) radical-scavenging	IC_50_ = 2.8 mg/L	Source of the *Origanum vulgare* L. and chemical constituents were mentioned above in reference [38].	[38]
	IC_50_ = 0.332 mg/mL (leaves flowers) IC_50_ = 0.357 mg/mL (roots) IC_50_ = 0.501 mg/mL (stems)	*Origanum vulgare* L. was collected from Huanggang City, China. Tested concentrations of OEO were: 0.10, 0.20, 0.40, 0.80, 1.60, and 3.20 mg/mL Qualitative and quantitative determination of the major phytocompounds were identified using GS-MS and attested the presence of: carvacrol (30.73%), thymol (18.81%), *p*-cymene (10.88%), caryophyllene (7.73%) in leaf-flower oils stem oils included large quantities of palmitic acid (60.18%), linoleic acid (14.25%), carvacrol (6.02%), thymol (3.46%), and oleic acid (5.65%) root oils included large quantities of palmitic acid (58.23%), linoleic acid (12.11%), linolenic acid (3.66%), carvacrol (3.27%), and thymol (1.08%).	[59]
	INU-CHI (inulin-chitosan) film containing 1.0%, 1.5%, 2% of combined *Origanum vulgare* L. and *Thymus vulgaris* L essential oils showed 38.79%, 42%, 57%, respectively DPPH radical scavenging activities	*Origanum vulgare* L. and *Thymus vulgaris* L. essential oils were incorporated into the INU-CHI (inulin-chitosan) in the concentration of 1.0%, 1.5%, and 2.0%. Essential oils were obtained from doTERRA (Pleasant Grove, UT, USA) Phytochemical constituents of the essential oils were not mentioned.	[60]
	DPPH scavenging activities: were 69.8 ± 0.8% for 2.5% OEO 87.5± 0.3% for 5% OEO 88.4 ±0.5% for 7.5% OEO	OEO was purchased from Edens Garden, San Clemente, CA, USA. OEO was encapsulated (2.5%, 5%, 7.5%) into nanofibres of PLCL/SF polymers. Phytochemical constituents of the essential oils were not mentioned.	[61]
	IC_50_ = 1057 μg/mL	*Origanum vulgare* L. was harvested from Gegharkunik province, Armenia. According to GC-MS assay the main constituents of the OEO were: *β*-caryophyllene epoxide (13.3%); *β*-caryophyllene (8.2%); *ο*-cymene (5.2%) The concentration of the OEO ranged from 1.95 to 1000 µg mL^−1^	[62]
Inhibition of Lipid Peroxidation by Ferric Thiocyanate assay (FTC)	At 5, 10 µg/mL OEO presented 23% and 38% lipid peroxidation inhibition. At 10 µg/mL, the inhibition percentage of the OEO was comparable to the standard (42% for 0.1 µg/mL of Trolox)	*Origanum vulgare* L. was collected from Chiang Mai, Thailand. According to GS-MS assay, the main constituents of the OEO were: carvacrol (79.5%), *γ*-terpinene (7.6%), *p*-Cymene (2.6%)	[63]
2,2′-Azino-Bis (3-Ethylbenzothiazoline)-6- Sulfonic acid (ABTS); radical scavenging	INU-CHI film containing 1.0%, 1.5%, 2% of the essential oils showed 83%, 90%, 98%, respectively ABTS radical scavenging activities	Source of the *Origanum vulgare* L. and chemical constituents were mentioned above in reference [60].	[60]
	At the concentration of 2 × 10^−3^% the TAS (the total antioxidant status) (2.20 ± 0.16 mmol/g) values increased by 307.4% in comparison with the negative control (TAS = 0.54 ± 0.04 mmol/protein	OEO was purchased from doTERRA International GS-MS attested the presence of: carvacrol (76.73%), thymol (11.34%), *p*-cymene (4.67%) The TAS(mmol/g protein) was determined by a chromogenic method (Randox Laboratories, UK) The protein concentrations were determined using the Bradford method. The total antioxidant status (TAS) levels of the OEO (8 × 10^−3^, 4 × 10^−3^, 2 × 10^−3^ *w*/*v*%) was tested on HaCaT (healthy human keratinocytes).	[64]

**Table 6:** Anti-inflammatory activity of *Origanum vulgare* L. essential oil (OEO).

Details: Source, Phytochemical Composition; Dose, Formulation	Mechanism of Action	Reference
*Origanum vulgare subsp. hirtum* EO was provided by Exentiae s.r.l. (Catania, Italy) Phytochemical constituents were not mentioned Nanostructured lipid carriers (NLC) loaded with OEO were obtained by phase inversion temperature and high-pressure homogenization, using two different emulsifiers systems (Tween80/Glyceryl oleate or Kolliphor RH40/Labrafil). Tested concentrations of NLC loaded with OEO were: 0.001, 0.002, 0.003% *v/v*	**decreases (↓)NO**	[66]
OEO was provided by Meritech Bioengineering Co. Ltd. (Guangzhou, China). According to GS-MS analysis, OEO contained the following major constituents: carvacrol (79.92%), thymol (1.90%), *γ*-terpinene (4.54%) OEO concentrations used in the experiment ranged from 2.5 to 10 μg/mL.	↓IL-1β, IL-6, TNF-α, ↓ ROS, inhibitory activity of NADPH oxidase	[67]
*Origanum vulgare* L. was harvested from Chaing Mai, Thailand. HPLC assay attested the presence of carvacrol (retention time = 3.381 min) as a major component of the OEO Transdermal microemulsion (ME) from OEO was tested IC_50_ = 6.8 μg/mL (↓ IL-6) and IC_50_ = 5.4 ± 2.3 (↓TNF-α)	↓IL-6, TNF-α	[68]
3 types of *Origanum vulgare* L. were harvested from Supra- Mediterranean and Meso- Mediterranean bioclimatic zones of Spain. OEO samples were analysed by GC-MS and the main constituents were: ***β***-Caryophyllene (0.5–4.9%), thymol (0.2–5.8%), ***p***-cymene (3.8–8.2%), ***γ***-terpinene (2.1–10.7%), carvacrol (58.7–77.4%) IC_50_ = 251.5 µL EO/L	inhibitory activity of lipoxygenase (LOX)	[69]

**Table 7:** In vitro anticancer activity of *Origanum vulgare* L. essential oil (OEO).

Details: Source, Phytochemical Composition; Dose, Formulation	Cancer Cell Line	Cell Proliferation, Apoptosis, Cytotoxicity, Dose, Incubation Time, Effect	Reference
*Origanum vulgare* L. was harvested from Chile. GS-MS assay of the OEO showed that 4-terpineol 41.17% was the major component, followed by thymol (21.95%), c-terpinene (5.91%), and carvacrol (4.71%). Cancerous cells were treated with the OEO ranging from 10 to 500 mg/mL.	human breast adenocarcinoma (MCF-7) human colon adenocarcinoma (HT-29)	The cytotoxicity test was performed by sulforhodamine B assay. Cancer cells were incubated for 72 h with OEO. The most effective concentration for HT-29(human colon adenocarcinoma) and MCF-7 (human breast adenocarcinoma) was 50 mg/mL and cell growth inhibition was 60.8% and 48.9%, respectively.	[71]
*Origanum vulgare* L. was collected from Basilicata Region, Southern Italy. According to GS-MS and GS-FID, thymol and carvacrol were the main phytocompounds (74.8%), followed by citral (2.5%). Tested concentrations ranged from 100 to 800 µg/µL.	hepatocarcinoma cell line (HepG2) non-tumour cell line (HEK293)	The cytotoxicity test was performed by MTT assay. They also analysed the cell morphology using phase-contrast microscopy. Cancer cells were incubated for 24 h with OEO IC_50_ = 236 µg/µL for HepG2 cells IC_50_ = 310 µg/µL for HEK293 cells HepG2 cells treated with OEO (236 µg/µL, for 24 h) showed morphological changes, such as detaching in the degradation phase.	[70]
OEO was purchased from Berjé USA. Chemical constituents of OEO were identified by GC–MS and consisted in: thymol (65.84%), p-cymene (9.86%), *γ*-terpinene (6.73%) Tested concentrations of OEO were: 5, 10, 25, 50 and 100 μg/mL.	human stomach cancer (AGS)	Antiproliferative property of OEO in AGS was determined by MTT assay. Cancer cells were incubated for 48 h with OEO. IC_50_ = 13.4 μg/mL The best antiproliferative activity of OEO was found to be 100 μg/mL.	[72]
Source of the material plant and chemical constituents were mentioned above in reference [38]. Tested concentrations of OEO were 50, 100, 200, 300, and 400 μg/mL	human breast adenocarcinoma (MCF-7) cervical adenocarcinoma (HeLa) T-cell lymphoblast (Jurkat) colon adenocarcinoma (HT-29) urinary bladder carcinoma (T24)	In comparison to positive controls (vinblastine sulfate and taxol) OEO presented significant antitumoral activities against: MCF-7 (IC_50_ = 8.11 µg/mL, taxol-IC_50_ = 0.08 µg/mL), HeLa (IC_50_ = 13.41 µg/mL, vinblastine-IC_50_ = 2.5 µg/mL) Jurkat (IC_50_ = 27.05 µg/mL, vinblastine-IC_50_ = 0,1 µg/mL) HT-29 (IC_50_ = 12.18 µg/mL, vinblastine-IC_50_ = 12.18 µg/mL) T24 (IC_50_ = 105.5 µg/mL, vinblastine-IC_50_ = 63.31 µg/mL)	[38]
OEO was purchased from Edens Garden, San Clemente, CA, USA. OEO was encapsulated (2.5%, 5%, 7.5%) into nanofibres of PLCL/SF polymers by electrospinning Phytochemical constituents of the essential oils were not mentioned	mammary carcinoma (mouse) 4T1 cell line	Cell viability was measured by cell counting kit-8 (CCK-8) assay. The incubation time for the cells on the material was 24, 48, and 72 h NF membranes with 5% and 7.5% OEO contents presented a strong antiproliferative effect after 72 h (*p* < 0,05)	[61]

**Table 8:** Beneficial effects of *Origanum vulgare* L. essential oil (OEO) on skin disorders.

Types of Skin Disorders	Details: Source, Phytochemical Composition; Dose, Formulation	Effects	Reference
Acne vulgaris	OEO was received from Nebraska University, Lincoln, NE, USA GS-MS assay determined the main phytocompounds: thymol (99.44%), *p*-cymene (0.2%), cineole (0.06%) OEO was formulated as nanoemulsion 5%(*w*/*w*) mixture of OEO and Pluronic F127 Nanoemulsion was tested in an acne animal mouse model (45 BALB/c, 6 weeks old, 20 g of weight) intradermally injected in ears with 20 µL of *Propionibacterium acnes*. They applied on infected mice ears, 20 µL of 2 MIC (1.34 mg/mL) oregano formulated nanoemulsion (test group), or 2% erythromycin (positive control), or no treatment (negative control) for 3 days.	Mice treated with nanoemulsion presented a higher anti-inflammatory response (>60%) compared to the positive control erythromycin (20%) The in vivo antimicrobial activity of OEO nanoemulsion was assessed where bacterial counts of *Propionibacterium acnes* dropped from 1 × 10^8^ to 4.3 × 10^1^ CFU (colony forming units)/mL post-treatment with 0.672 mg/mL of oregano nanoemulsion. -Histopathological and digital photography of infected mice ears treated revealed normal histology of mouse ear tissue with the absence of inflammatory reaction.	[39]
Wound and anti-inflammatory potential	OEO was provided by Esperis S.p.A., Milan, Italy. The qualitative and quantitative characterization of OEO was assessed by GC and GC-MS. The main components were: carvacrol (35.95 + 0.22%), thymol (25.2 + 0.27%), *p*-cymene (21.54 + 0.35%), linalool (4.26 + 0.05%). Different concentrations of OEO (3, 5, 7.5, 10, 12, 25, and 50 μg/mL were tested but 25 μg/mL concentration was used as a standard for all experiments. Antioxidant activity was evaluated in NCTC 2544 cells line measuring the 2′,7′ oxidation dichlorodihydrofluorescein diacetate and the incubation time was 72 h Analysis regarding the amount of mRNA and proteins of ICAM-1, iNOS, and COX-2 were assessed by RT-PCR and Western blot PCNA modulation was evaluated by immunoblot human keratinocyte cell line NCTC 2544 was used as an inflammatory in vitro model.	reduction and/or modulation of the inflammatory parameters: ROS(reactive oxygen species), ICAM-1(intracellular cell adhesion molecule 1) -iNOS(inducible nitric oxidesynthase) -COX_2_ MMP-1 (matrix metalloproteinase 1)and MMP-12 PCNA(proliferating cell nuclear antigen)	[73]
	OEO was provided by doTERRA, Pleasant Grove, UT, USA Phytochemical constituents were not mentioned Dermal fibroblast system was designed to model chronic inflammation and fibrosis (BioMAP HDF3CGF) Cell proliferation and viability of four concentrations of OEO (0.011, 0.0037, 0.0012, and 0.00041%, *v/v*) were measured using a sulforhodamine B assay The potential anti-inflammatory and tissue remodelling properties of the OEO were tested via ELISA method.	The research group showed that the concentration (0.011% *v/v*) was cytotoxic Results showed a significant antiproliferative activity to dermal fibroblast cells. OEO (0,0037% *v/v* OEO) significantly decreased the levels of inflammatory biomarkers such as MCP-1 (monocyte chemoattractant protein 1), VCAM-1 (vascular cell adhesion molecule 1), ICAM-1 (intracellular cell adhesion molecule 1), IP-10 (Interferon gamma-induced protein 10), I-TAC (interferon-inducible T-cell alpha chemoattractant), and MIG (monokine induced by gamma interferon). Moreover, OEO diminished tissue remodelling biomarkers such as collagen I, collagen III, M-CSF (macrophage colony-stimulating factor), EGRF (epidermal growth factor receptor), MMP-1, PAI-1, TIMP-1 (tissue inhibitor of metalloproteinase-1), and TIMP- 2.	[74]
Skin aging	Whole plants of *Origanum vulgare* L. were harvested from Chiang Mai, Thailand Commercial OEO was obtained from Botanicessence (Product of Spain). GS-MS chromatograms noted carvacrol, *m*-thymol, *p*-cymene, and *γ*-terpinene as major chemical constituents in both oils. For the determination of anti-collagenase, anti-elastase activity, anti-hyaluronidase activity were used ascorbic acid and oleanolic acid as positive controls.	OEO obtained from a tropical area in Thailand had greater anti-skin-aging activity than ascorbic acid (*p* < 0.01), and the inhibitory activities against collagenase, elastase, and hyaluronidase were 92.0 ± 9.7%, 53.1 ± 13.3%, and 16.7 ± 0.3%, at the concentration of 67, 25, and 4 µg/mL, respectively. The anti-hyaluronidase activity of both essential oils (commercial OEO-15.5% and OEO from Chiang Mai-16.7%) was more mitigated than the one of oleanolic acid (86%).	[63]

## Abbreviations

2D-SDS-PAGE	Two-dimensional sodium dodecyl sulfate–polyacrylamide gel electrophoresis
ABTS	2,2′-Azino-Bis (3-Ethylbenzothiazoline)-6- Sulfonic acid
AChE	Acetylcholinesterase
AGS	Adenocarcinoma gastric cell line
BAX	Apoptosis regulator
BChE	Butyrylcholinesterase
BCL-2	B-cell lymphoma protein 2
bio-AgNP	Green silver nanoparticles
CCK-8	Cell counting kit-8
CFU	Colony forming units
CO	Commercial oil from the Mediterranean region
CZD	Clear zone diameter
DNA	Deoxyribonucleic acid
EGRF	Epidermal growth factor receptor
ELISA	Enzyme-linked immunosorbent assay
EO	Essential oil
ESBL	Extended-spectrum beta-lactamase
FGC/MS	Fast gas chromatography/mass spectrometry
GC-MS/FID	Gas chromatography-mass spectrometry/flame ionization detector
GC-MS	Gas chromatography-mass spectrometry
H2DCFDA	2′,7′-dichlorodihydrofluorescein diacetate
HaCaT	Healthy human keratinocytes
HCT-8 cells	Human colon adenocarcinoma
HeLa	Cervical adenocarcinoma
HepG2	Hepatocarcinoma cell line
HO	Essential oil from a highland area of a tropical country
HT-29	Human colon adenocarcinoma
IC_50_	The half maximal inhibitory concentration
ICAM-1	Intracellular cell adhesion molecule 1
IFN-γ	Interferon-gamma
IL-6	Interleukin-6
iNOS	Inducible nitric oxidesynthase
INU-CHI	Inulin-chitosan
IP-10	Interferon gamma-induced protein 10
IS	Irritation score
I-TAC	Interferon-inducible T-cell alpha chemoattractant
Jurkat	T-cell lymphoblast
KAEs	Kojic acid equivalents
KPC	*K. Pneumoniae* carbapenemase
LOX	Lipoxygenase
MBC	Minimum bactericidal concentration
MBEC	Minimal biofilm eradication concentration
MBIC	Minimum biofilm inhibitory concentration
MCF-7	Human breast adenocarcinoma
MCP-1	Monocyte chemoattractant protein 1
M-CSF	Macrophage colony-stimulating factor
MDR	Multidrug-resistant
ME	Microemulsion of *O. Vulgare* essential oil
MFC	Minimum fungicidal concentration
MIC	Minimum inhibitory concentration
MIG	Monokine induced by gamma interferon
MLC	Minimal lethal concentration
MMP-1	Matrix metalloproteinase 1
MMP-2	Matrix metalloproteinase 2
mRNA	Messenger Ribonucleic acid
MRSA	Methicillin-resistant *S. Aureus*
MtP	Microtiter-plate test
MTT	3-(4,5-dimethylthiazol-2-yl)-2,5-diphenyl tetrazolium bromide
NADPH	Nicotinamide adenine dinucleotide phosphate
NCTC 2544	Human keratinocyte cell line
NF	Nanofibrous membrane
NLC	Nanostructured lipid carriers
NO	Nitric oxide
OEO	*Origanum vulgare* L. essential oil
OT	Oregano and thyme essential oils
OVH	*Origanum vulgare subsp.hirtum*
OVV	*Origanum vulgare subsp. Vulgare*
PAI-1	Plasminogen activator inhibitor 1
PCNA	Proliferating cell nuclear antigen
PI	Propidium iodide
PLCL/SF	Poly l-lactic acid- co-e-caprolactone/Silk Fibroin
PZ	precipitation zone
RAW 264.7 cells	Murine macrophage cell line
ROS	Reactive oxygen species
SEM	Scanning electron microscope
T24	Urinary bladder carcinoma
TAS	The total antioxidant status
TEM	Transmission electron microscopy
TIMP-1	Tissue inhibitor of metalloproteinase
TNF-α	*Tumor necrosis factor*
TUNEL assay	Terminal deoxynucleotidyl transferase dutp nick end labeling
VCAM-1	Vascular cell adhesion molecule 1
